# Social processes as the missing link: cross-sectionally testing a conceptual model on social mediators of early psychopathological development

**DOI:** 10.1017/S0033291724001594

**Published:** 2024-10

**Authors:** Robin Achterhof, Olivia J. Kirtley, Ginette Lafit, Anu P. Hiekkaranta, Noëmi Hagemann, Karlijn S. F. M. Hermans, Aleksandra Lecei, Bart Boets, Cécile Henquet, Maude Schneider, Rob Sips, Thomas Vaessen, Ruud van Winkel, Wolfgang Viechtbauer, Ulrich Reininghaus, Inez Myin-Germeys

**Affiliations:** 1 Center for Contextual Psychiatry, Research Group Psychiatry, Department of Neurosciences, KU Leuven, Leuven, Belgium; 2 KU Leuven Child & Youth Institute, KU Leuven, Leuven, Belgium; 3 Erasmus School of Social and Behavioural Sciences, Department of Psychology, Education & Child Studies, Erasmus University Rotterdam, Mandeville Building Room T15-10, P.O. Box 1738, 3000 DR Rotterdam, The Netherlands; 4 Research Group on Quantitative Psychology and Individual Differences, Faculty of Psychology, KU Leuven, Leuven, Belgium; 5 Flemish Scientific Society for Youth Health Care (VWVJ), Leuven, Belgium; 6 Strategy and Academic Affairs, Administration and Central Services, Leiden University, Leiden, The Netherlands; 7 Center for Clinical Psychiatry, Research Group Psychiatry, Department of Neurosciences, KU Leuven, Leuven, Belgium; 8 Center for Developmental Psychiatry, Research Group Psychiatry, Department of Neurosciences, KU Leuven, Leuven, Belgium; 9 Open University of the Netherlands, Heerlen, the Netherlands; 10 Clinical Psychology Unit for Intellectual and Developmental Disabilities, Faculty of Psychology and Educational Sciences, University of Geneva, Geneva, Switzerland; 11 Center for eHealth and Well-being Research, Department of Psychology, Health, and Technology, University of Twente, Enschede, The Netherlands; 12 Department of Public Mental Health, Central Institute of Mental Health, Medical Faculty Mannheim, Heidelberg University, Mannheim, Germany; 13 ESRC Centre for Society and Mental Health and Centre for Epidemiology and Public Health, Health Service and Population Research Department, Institute of Psychiatry, Psychology & Neuroscience, King's College London, London, UK

**Keywords:** adolescents, bullying, psychopathology, social skills, social support, trauma

## Abstract

**Background:**

Research suggests that most mental health conditions have their onset in the critically social period of adolescence. Yet, we lack understanding of the potential social processes underlying early psychopathological development. We propose a conceptual model where daily-life social interactions and social skills form an intermediate link between known risk and protective factors (adverse childhood experiences, bullying, social support, maladaptive parenting) and psychopathology in adolescents – that is explored using cross-sectional data.

**Methods:**

*N* = 1913 Flemish adolescent participants (Mean age = 13.8; 63% girls) were assessed as part of the SIGMA study, a large-scale, accelerated longitudinal study of adolescent mental health and development. Self-report questionnaires (on risk/protective factors, social skills, and psychopathology) were completed during class time; daily-life social interactions were measured during a subsequent six-day experience-sampling period.

**Results:**

Registered uncorrected multilevel linear regression results revealed significant associations between all risk/protective factors and psychopathology, between all risk/protective factors and social processes, and between all social processes and psychopathology. Social processes (social skills, quantity/quality of daily social interactions) were uniquely predicted by each risk/protective factor and were uniquely associated with both general and specific types of psychopathology. For older participants, some relationships between social processes and psychopathology were stronger.

**Conclusions:**

Unique associations between risk/protective factors and psychopathology signify the distinct relevance of these factors for youth mental health, whereas the broad associations with social processes support these processes as broad correlates. Results align with the idea of a social pathway toward early psychopathology, although follow-up longitudinal research is required to verify any mediation effect.

The foundations of adult mental health are laid early on, as the majority of all mental health conditions have their onset during adolescence (Kessler et al., 2005; Merikangas et al., 2010; Solmi et al., 2021). Estimates indicate that 22% of adolescents present with severe and distressing mental health disorders, and that this prevalence is higher among girls than boys (Merikangas et al., 2010). Mental health problems in adolescence are associated with numerous negative clinical, functional, and sociodemographic outcomes (Asselmann, Wittchen, Lieb, & Beesdo-Baum, 2018), including future suicide attempts (Miché et al., 2018). Furthermore, the younger a person is when they first develop a mental health disorder, the more years they have to live with this, and the higher the chances of developing comorbid disorders later on (Caspi et al., 2020). Adolescence thus presents a unique window of opportunity for prevention and early intervention programs (Colizzi, Lasalvia, & Ruggeri, 2020).

The development of mental health problems has been associated with a wide array of transdiagnostic risk and protective factors that, as such, predict a range of different psychopathologies (e.g. depression, anxiety). Many identified transdiagnostic factors have a social component, including low social support (Dalgard, Bjork, & Tambs, 1995), exposure to bullying (Brendgen, 2018), family problems (Davies & Sturge-Apple, 2014), exposure to adverse childhood experiences or trauma (McKay et al., 2021), and growing up in an urban environment (Polanczyk et al., 2010). In adolescence in particular, social risk and protective factors seem to play a large role in predicting a range of mental health outcomes (Klasen et al., 2015; Scardera et al., 2020). Furthermore, what we know relatively little about, is *how* these risk/protective factors may produce adaptive *v.* maladaptive development. To aid prevention and early intervention efforts, we need to learn more about the potential underlying processes through which these risk and protective factors are associated with mental health outcomes.

In the current paper, we propose that these underlying processes are also fundamentally *social* in nature. Risk and protective factors may shape how adolescents interact with others (i.e. they affect social processes), which, in turn, drives the dynamic process of psychopathological development. Following leading theories of developmental psychopathology (Cicchetti & Toth, 1997), we consider that psychopathology arises in conjunction with altered dynamic interactions between individuals and their (social) context. As such, the simplest depiction of this idea is represented in the mediation model of Fig. 1, where social processes mediate the relationship between risk/protective factors and psychopathology, but also, where psychopathology impacts those same social processes. Although formally testing this mediation requires longitudinal data (Kline, 2015), more detailed insight into these cross-sectional associations can provide a solid empirical basis for further investigations.

**Figure 1.**
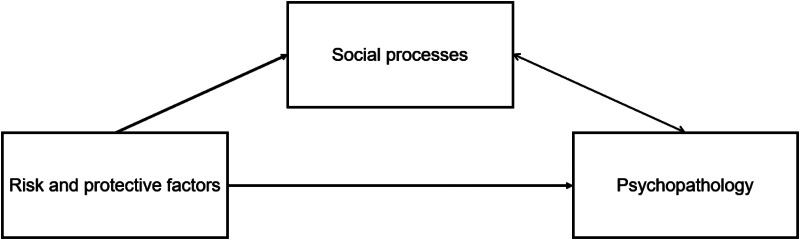
Implied mediation model underlying the reciprocal associations between social processes and psychopathology, and the effects of risk/protective factors.

The social nature of psychopathological development is already supported, for example, by findings of diminished interpersonal skills across different types of mental health issues (Segrin, 2019). At the same time, much research is hampered by a lack of ecological validity – or the real-world representativeness and generalizability of these social drivers of development. Traditional measures of interpersonal skills may not suffice for comprehensively capturing the social processes that link risk/protective factors to psychopathology (Hermans et al., 2019). If we aim to better understand how different social processes are related to early psychopathological development, we need to take a more direct look at the proximal social processes relating to *daily-life social interaction*. Previous research drawing on the experience sampling method (ESM; Csikszentmihalyi & Larson, 1987; Myin-Germeys et al., 2018) has already identified associations between both the quantity and quality of daily social interactions, and different types of adolescent psychopathology (Achterhof et al., 2021). Yet, it remains unclear to what extent these alterations in different social processes link to key risk and protective factors.

Moreover, as adolescence is a critical period of both changes in one's relationships and in one's mental health (Blakemore & Mills, 2014; Orben, Tomova, & Blakemore 2020), we can also expect age-related differences in the contribution of different social processes to the development of psychopathology. A fundamental task of adolescence is to gain a better understanding of oneself in relation to others (Grusec & Davidov, 2010; Smetana, Robinson, & Rote, 2015), which is accompanied by an increasing social network outside of the family (Wrzus, Hänel, Wagner, & Neyer, 2013). To do achieve this task, sufficient interpersonal skills and qualitative social interactions may become more important as adolescents age. In addition, as boys and girls tend to differ both in terms of mental health (Campbell, Bann, & Patalay, 2021) and social development (Smetana, Campione-Barr, & Metzger, 2006), we can expect gender differences in the role of social processes to psychopathological development. In particular, recent evidence suggests that social interaction quality is more important for adaptive development in girls than boys (Barzeva, Richards, Veenstra, Meeus, & Oldehinkel, 2022).

The SIGMA study has been set up specifically to investigate both risk and protective factors as well as naturalistic social processes in a general population adolescent sample in Flanders, Belgium (Kirtley et al., 2021). Social processes were assessed both using a self-report questionnaire on social skills, as well as a structured diary technique, the Experience Sampling Method (ESM), providing a unique opportunity to assess in daily life both the quantity of social interactions (how often adolescents were in interaction with others) as well as the quality of these social interactions (how adolescents experienced the social interactions; Achterhof et al., 2022a). By including different age cohorts of adolescents from the outset of this longitudinal study (i.e. an accelerated longitudinal design; Galbraith, Bowden, & Mander, 2017), we are able to test potential developmental effects with data from the first wave. Although the proposed model suggests reciprocal effects between social processes and psychopathology (see Fig. 1), we acknowledge that the data used in this study does not allow for formally testing such effects (Maxwell & Cole, 2007). Risk and protective factors were selected based on their previous robust associations with mental health outcomes (e.g. Brendgen, 2018; Davies & Sturge-Apple, 2014; McKay et al., 2021). They mostly refer to past events and experiences; however, as these questionnaires have not been completed at a preceding time point, we also acknowledge that we are unable to formally test the directionality of their effects. Instead, our current aim is to implicitly test the plausibility of this model by assessing all individual effects contained within it.

As such, the current study uses the data of the first wave of the SIGMA study to investigate (1) the prevalence of mental health problems in this large general population sample as well as the role of age and gender, (2) the associations between known risk and protective factors (adverse childhood experiences, bullying, parental psychological control, social support) and mental health problems (general, depression, anxiety, psychoticism), (3) the associations between known risk and protective factors and social processes (interpersonal skills, the quantity and quality of daily-life social interactions), (4) the associations between social processes and mental health problems. Both corrected and uncorrected associations will be tested throughout. Finally, it will be investigated whether the associations as outlined in 1–4, change with increasing age. As such, this study serves as a test case of different transdiagnostic social pathways toward the development of early mental health problems.

## Methods

### Recruitment and sample

The SIGMA study recruited *N* = 1913 adolescents in 22 schools across Flanders, Belgium (for detailed info, see Kirtley et al., 2021), between January 2018 and June 2019. Adolescents were eligible to participate if they were in the 1st, 3rd, or 5th grade of secondary school. Participants and their parents were asked to provide informed assent and consent, respectively, for study participation. The study was approved by the Leuven Medical Ethical Committee (number S 61395).

### Procedure

Participants were tested during a 100-min in-class session, in groups of 10–25 students. Participants filled in a battery of questionnaires individually on tablets, using the REDCap application (Harris et al., 2009), with the researchers present. Afterwards, participants received a study phone and were instructed for the ESM part of the study. Every day for the next six days, participants were prompted ten times per day to complete the ESM questionnaires on a research mobile phone with only the ESM application ‘MobileQ’ pre-installed (Meers, Dejonckheere, Kalokerinos, Rummens, & Kuppens, 2020). Questionnaire prompts were randomly distributed within each of ten 90-min blocks per day between 7.30 AM and 10.30 PM, with at least 15 min between consecutive prompts. Participants had 90 s to respond to each prompt, and 15 min to complete each ESM questionnaire once opened. As compensation for full study participation, adolescents received a €10 euro voucher for an online store.

### Measures

#### Psychopathology symptoms

**Psychopathology** was assessed with the Brief Symptom Inventory (BSI; Derogatis, 1993), consisting of 53 items across nine subscales rated on a scale from 0 (not at all) to 4 (very much). The Global Severity Index (GSI) is the average response to all items. Reliability of the GSI was good, as assessed with both Cronbach's alpha (*α* = 0.96) and McDonald's Omega (Revelle & Condon, 2019; *Ω* = 0.97). The subscales Depression (*α* = 0.88; *Ω* = 0.90), Anxiety (*α* = 0.78; *Ω* = 0.87) and Psychoticism (*α* = 0.73; *Ω* = 0.75) consisted of the mean scores on those subscales.

#### Risk and protective factors

**Adverse Childhood Experiences (ACE)** were assessed with the Juvenile Victimization Questionnaire, revision 2 (JVQ-R2; Hamby, Finkelhor, Ormrod, & Turner, 2004). With 34 yes/no items, four types of traumatic events were assessed: property crime, physical assault, sexual assault, and general maltreatment. Nine items of the ‘conventional crime module’ (e.g. relating to firearms) were considered of limited relevance for this sample and were removed for all first-grade students. All analyses were performed with a total score without this module, but a set of sensitivity analyses was performed with the full JVQ-R2 score as well (see online Supplementary Material S1). ACE was defined as the total number of adverse events reported by the participant (reliability of total score: *α* = 0.86; *Ω* = 0.87).

**Bullying** was assessed with one item inquiring about prevalence of bullying (*‘Has another kid or teenager ever physically hurt you?* (*e.g. burned, bruised, cut, punched, kicked*)*’*) with answer options ‘Never’, ‘Rarely’, ‘Sometimes’, ‘Regularly’ and ‘Often’. An additional item inquired about the severity of the bullying (‘How bad was the bullying?), with answer options ‘Not, ‘A little’, ‘Moderately, and ‘Severely’. The product of both items was taken as the overall bullying score.

**Parental psychological control** was assessed for each parent by taking the mean of the eight items of the Psychological Control Scale – Youth Self-Report (Barber, 1996), all ranging from ‘1. Not at all’ to ‘5. Very much’ (e.g. ‘*My mother/father is always trying to change how I feel or think about things*’, *α* = 0.84; *Ω* = 0.87).

**Social support** was assessed with the Dutch Social Support List (Van Sonderen, 2012), consisting of 12 items worded with ‘*Does it ever happen you that people*…’ and divided into three subscales: everyday support (e.g. ‘*Show you that they are fond of you?*’), support in case of problems (e.g. ‘*Comfort you*?’), and appreciation (e.g. ‘*Emphasize your strong points?’*). Items were rated on a ‘1. Rarely or never’ to ‘4. Very often’ scale. A total mean score was calculated, with higher scores indicating more social support (*α* = 0.85; *Ω* = 0.87).

#### Social processes

**Interpersonal skills** were assessed with the Dutch self-report ‘Questionnaire Psychosocial Skills’ (VPV; (Scholte & Van der Ploeg, 2013). The VPV consists of the two subscales ‘Interpersonal Skills’ and ‘Intrapersonal Skills’ – only the former subscale was used. This subscale, in turn, consists of two subscales on ‘Relational Skills’ (e.g. ‘*I seek out to connect with peers that I like*’) and ‘Affective Skills’ (e.g. ‘*I recognize in others how they feel or think*’), each consisting of nine items rated on a scale ranging from ‘1. Completely disagree’ to ‘5. Completely agree’. We used the full ‘Interpersonal Skills’ subscale, which showed good reliability (*α* = 0.83; *Ω* = 0.86).

#### Experience sampling methodology

To obtain the moment-level indicators of quantity/quality of daily social interactions, we draw on the social interaction data obtained throughout our six-day ESM period. The full ESM questionnaire is included in online Supplementary Material S2, and is also available in the online ESM Item Repository (www.esmitemrepository.com; Kirtley et al., 2020).

***Quantity of Social Interactions.*** A dichotomous moment-level ‘Social Interaction’ score was constructed by taking the ESM prompts where participants indicated being both in the company of others (as indicated by any company type as a response to the item ‘Who am I with?’) and interacting with them (as indicated by at least a ‘2’ on the item ‘We are doing something together’). As the person-mean level of social interactions is predicted in each multilevel logistic regression model, we label this variable ‘Quantity of Social Interactions’.

***Quality of Social Interactions.*** If participants indicated company in the ESM questionnaire, three qualitative items followed (‘I feel comfortable in this company’, ‘I feel valued in this company’, ‘I feel like I belong’), all rated from ‘1. Not at all’ to ‘7. Very much’. A mean ‘Quality of Social Interactions’ score was computed by taking the moment-level mean of these three items. Note that, strictly speaking, these items assess the *experienced* quality of social interaction at each time point – only as assessed by the individual participant. Within-person reliability for this variable was *ω*_within_ = 0.86; between-person reliability was *ω*_between_ = 0.92.

### Statistical analysis

#### Sample size and statistical power

Prior to data collection, a simulation-based sample size calculation was conducted for the relatively complex research question on affective reactivity to the environment, which resulted in an estimated power of 0.94 for a total *n* = 2.001 (see Kirtley et al., 2021), for more details). For the relatively simpler linear regression analyses included in this study, a sensitivity power calculation was performed in G × Power, to calculate the minimal effect size that we are able to find. This demonstrated that a sample size of 1913 would be able to detect at least a small (f2 = 0.01) effect size with 90% power, *α* = 0.05 and 6 predictors (4 tested + 2 covariates) in a multiple linear regression analysis.

#### Missing data handling

For the missing data on all (non-ESM) questionnaires, we imputed data at the item-level, based on the information of all other variables, using a multiple imputation model by chained equations (MICE), with the ‘mice’-package *v.* 3.14.0 (van Buuren & Groothuis-Oudshoorn, 2011) in R. All estimates were obtained by pooling the results (using Rubin's rules; Rubin, 1987) of each performed analysis on the 20 imputed datasets.

#### Analysis strategy

First, basic descriptive statistics were computed. The investigation of age and gender effects was performed by including these variables as covariates within the following models.

Second, associations between risk/protective factors and mental health outcomes were estimated in linear regression models estimating the effects of each of four risk/protective factors on each of four psychopathology outcomes. Corrected models included all risk/protective factors simultaneously and one psychopathology outcome; uncorrected models included one risk/protective factor per model.

Third, the associations between all risk/protective factors and interpersonal functioning (the first of three social processes) were estimated similarly: predicting interpersonal functioning in corrected and uncorrected linear regression models with the risk/protective factors as predictors. As the quantity and quality of social interaction variables are moment- (rather than person-)level variables, they necessitated a multilevel approach. Logistic multilevel regression models were estimated to assess the effect of each risk/protective factor on the probability of engaging in a social interaction at any given time point. Linear multilevel regression models were estimated to assess the effect of each risk/protective factor on the mean social interaction quality at any given time point.

Fourth, we needed to consider that, in these multilevel models, it is impossible to predict a Level 2-outcome (i.e. psychopathology) from a Level-1 predictor (i.e. quantity/quality of social interaction) (Preacher, Zyphur, & Zhang, 2010). As we were interested in cross-sectional associations rather than any temporal (let alone causal) ordering of effects, we considered it appropriate to estimate the effect of each psychopathology variable on the quantity/quality of social interactions – rather than vice versa. We estimated the effects of each psychopathology variable on the quantity of social interactions in logistic multilevel regression models, and on the quality of social interactions in linear multilevel regression models. We also estimated the effect of psychopathology on interpersonal functioning in a set of linear regression models.

Finally, to assess differential effects according to age, interactions between age and each relevant predictor were added to all uncorrected models described above.

### Open science practices

All hypotheses and the full analysis plan for the study were post-registered – a type of pre-registration occurring after data collection, but before data access or analysis (Benning, Bachrach, Smith, Freeman, & Wright, 2019) – on the Open Science Framework (OSF; https://osf.io/jhav7/?view_only=74cf9b5ba7924e35ba7d7bcd02dbd812). The limited deviations from the registration are listed in online Supplementary Material S3. For additional transparency, all used R code and output is made available online (osf.io/6h7be/).

## Results

### Descriptive statistics

Descriptive statistics of all included variables are listed in Table 1 (all reflecting the imputed data; see online Supplementary Material S4 for descriptive statistics of raw data). *N* = 1913 adolescents (*n* = 1214 girls) participated in the study. Seven participants indicated their gender as other than male or female; gender information was missing for four participants. Three age cohorts were included, from three different years of Flemish secondary school: 1st year students (~ 12 years old; *n* = 1048), 3rd year students (~14 years old; *n* = 424), and 5th year students (~16 years old; *n* = 441). Compliance to the ESM protocol was relatively low, with, on average, 40% (s.d. = 22.8) of daily questionnaires answered by participants.

**Table 1.** Descriptive characteristics of full sample, pooled across the 20 multiple imputed datasets

	Full sample (*n* = 1913)		
Variables	Mean (s.d.)	Median	Range
Age	13.8 (1.9)	13.0	11.0–20.0
Gender (%girls)	63.4		
First grade (%)	54.8		
Third grade (%)	22.2		
Fifth grade (%)	23.1		
Parental psychological control	2.0 (0.6)	1.8	1.0–4.7
Social support	1.9 (0.5)	1.9	0.0–3.0
Bullying	1.9 (3.1)	1.0	0.0–12.0
Adverse childhood experiences	4.1 (3.4)	3.0	0.0–24.0
Interpersonal skills	3.9 (0.4)	3.8	1.0–5.0
Mean quantity of social interactions, as % of all compliant beeps	70.6 (20.7)	73.9	0.0–100.0
Mean quality of social interactions	5.8 (1.0)	6.0	1.0–7.0
Depression	0.8 (0.8)	0.5	0.0–4.0
Anxiety	0.9 (0.7)	0.7	0.0–4.0
Psychoticism	0.7 (0.7)	0.4	0.0–3.3
Psychopathology – total (GSI)	0.9 (0.6)	0.7	0.0–3.4

Correlations between all included continuous variables are presented in Table 2. Correlations between all psychopathology variables are generally quite high, ranging from 0.67 to 0.82 for the correlations between depression, anxiety, and psychoticism.

**Table 2.** Pearson correlations between all included (person-level) variable, pooled across the 20 multiple imputed datasets

	PPC	Social support	Bullying	ACE	Interpersonal skills	Quantity of SI^a^	Quality of s.e.^a^	Depression	Anxiety	Psychoticism	GSI
Age	0.22**	−0.09**	0.10**	0.24**	−0.12**	−0.09**	−0.16**	0.24**	0.13**	0.19**	0.20**
PPC		−0.21**	0.20**	0.44**	−0.21**	−0.13**	−0.34**	0.40**	0.35**	0.40**	0.46**
Social support			−0.14**	−0.15**	0.54**	0.12**	0.34**	−0.24**	−0.10**	−0.20**	−0.19**
Bullying				0.38**	−0.09**	−0.08*	−0.20**	0.38**	0.29**	0.32**	0.37**
ACE					−0.20**	−0.14**	−0.30**	0.43**	0.40**	0.44**	0.51**
Interpersonal skills						0.11**	0.34**	−0.26**	−0.15**	−0.22**	−0.25**
Quantity of SI^a^							0.25**	−0.18**	−0.11**	−0.17**	−0.17**
Quality of SI^a^								−0.37**	−0.25**	−0.32**	−0.36**
Depression									0.67**	0.82**	0.87**
Anxiety										0.69**	0.85**
Psychoticism											0.87**

*Note*: PPC, Parental Psychological Control; ACE: Adverse Childhood Experiences; GSI, General Severity Index (total psychopathology score).

a The quantity and quality of social interaction variables represent person-level aggregates representing person means.

*p < 0.01; **p < 0.001.

### Age, gender and psychopathology

As age and gender were included as covariates in the prediction of outcomes of psychopathology, we can see in Table 3 how – for general psychopathology, depression, anxiety, and psychoticism – girls reported significantly more symptoms than boys. Also, compared to younger participants, older participants reported significantly more depressive symptoms, but there was no significant relationship between age and general psychopathology, psychoticism or anxiety, after correcting for multiple testing.

**Table 3.** Associations between risk/protective factors and psychopathology

	Outcomes															
	GSI				Depression				Anxiety				Psychoticism			
	Corrected		Uncorrected		Corrected		Uncorrected		Corrected		Uncorrected		Corrected		Uncorrected	
Predictors	*β* (s.e.)	*p*	*β* (s.e.)	*p*	*β* (s.e.)	*p*	*β* (s.e.)	*p*	*β* (s.e.)	*p*	*β* (s.e.)	*p*	*β* (s.e.)	*p*	*β* (s.e.)	*p*
Intercept	0.75 (0.02)	**<0.001**	–		0.62 (0.03)	**<0.001**	–		0.77 (0.02)	**<0.001**	–		0.57 (0.02)	**<0.001**	–	
Age	0.01 (0.01)	0.08	–		0.04 (0.01)	**<0.001**	–		–−0.00 (0.01)	0.81	–		0.02 (0.01)	*0*.*024*	–	
Sex (ref = male)	0.20 (0.02)	**<0.001**	–		0.27 (0.03)	**<0.001**	–		0.20 (0.03)	**<0.001**	–		0.16 (0.03)	**<0.001**	–	
PPC	0.24 (0.02)	**<0.001**	0.41 (0.02)	**<0.001**	0.26 (0.03)	**<0.001**	0.45 (0.03)	**<0.001**	0.22 (0.03)	**<0.001**	0.36 (0.03)	**<0.001**	0.24 (0.03)	**<0.001**	0.40 (0.02)	**<0.001**
Social support	−0.10 (0.02)	**<0.001**	−0.22 (0.03)	**<0.001**	−0.23 (0.03)	**<0.001**	−0.38 (0.04)	**<0.001**	−0.01 (0.03)	0.66	−0.13 (0.03)	**<0.001**	−0.13 (0.03)	**<0.001**	−0.26 (0.03)	**<0.001**
Bullying	0.03 (0.00)	**<0.001**	0.06 (0.00)	**<0.001**	0.05 (0.01)	**<0.001**	0.09 (0.01)	**<0.001**	0.03 (0.01)	**<0.001**	0.06 (0.01)	**<0.001**	0.03 (0.01)	**<0.001**	0.06 (0.01)	**<0.001**
ACE	0.06 (0.00)	**<0.001**	0.09 (0.00)	**<0.001**	0.06 (0.01)	**<0.001**	0.09 (0.01)	**<0.001**	0.05 (0.01)	**<0.001**	0.08 (0.00)	**<0.001**	0.05 (0.01)	**<0.001**	0.08 (0.00)	**<0.001**

PPC, parental psychological control; GSI, General Severity Index (general psychopathology); ACE, Adverse Childhood Experiences.

Corrected models include all variables in that column simultaneously as predictors; uncorrected models include age, sex, and each risk/protective factor separately as predictor.

Since each column with uncorrected associations represents four separate models with different values for intercept, age, and sex, the cells representing those values are empty in this table (see full model output online for coefficients, osf.io/6h7be/).

*Note*: *p* values in bold indicate a significant effect following Holm's multiple comparison correction with initial *α* = 0.05; *p* values in italics indicate a significant effect after *α* = 0.05.

### Risk/protective factors and psychopathology

All four risk and protective factors were significantly associated with psychopathology in four separate models, both in the model of general psychopathology as well as in the models of anxiety, depression, and psychoticism (Table 3, uncorrected models). Higher levels of ACE, bullying and psychological parental control as well as lower levels of social support were associated with higher levels of psychopathology. When all predictors were entered into one model, associations remained significant (Table 3, corrected models), except for the association between social support and anxiety symptoms.

### Risk/protective factors and social processes

All four risk and protective factors were significant in the models predicting the three social processes (Table 4, uncorrected models). So, higher levels of ACE, bullying, psychological parental control and lower levels of social support were associated with fewer interpersonal skills, and a lower quantity and quality of social interactions. When predictors were entered simultaneously, their unique contributions remained significant (Table 4, corrected models), except for the associations between bullying and interpersonal skills, and between bullying and quantity of social interactions.

**Table 4.** Associations between risk/protective factors and social processes

	Outcomes											
	Interpersonal skills				Quantity of SI				Quality of SI			
	Corrected		Uncorrected		Corrected		Uncorrected		Corrected		Uncorrected	
Predictors	*β* (s.e.)	*p*	*β* (s.e.)	*p*	*β* (s.e.)	*p*	*β* (s.e.)	*p*	*β* (s.e.)	*p*	*β* (s.e.)	*p*
Intercept	3.84 (0.01)	**<0.001**			1.02 (0.04)	**<0.001**			5.78 (0.03)	**<0.001**		
Age	−0.01 (0.00)	*0*.*028*			−0.04 (0.01)	**0.010**			−0.03 (0.01)	*0*.*031*		
Sex (ref = male)	0.03 (0.02)	0.17			0.03 (0.05)	0.53			−0.02 (0.04)	0.68		
PPC	−0.04 (0.02)	**0.015**	−0.13 (0.02)	**<0.001**	−0.13 (0.05)	**0.008**	−0.24 (0.04)	**<0.001**	−0.31 (0.04)	**<0.001**	−0.51 (0.04)	**<0.001**
Social support	0.43 (0.02)	**<0.001**	0.45 (0.02)	**<0.001**	0.21 (0.05)	**<0.001**	0.27 (0.05)	**<0.001**	0.55 (0.04)	**<0.001**	0.67 (0.05)	**<0.001**
Bullying	0.00 (0.00)	0.22	−0.01 (0.00)	**0.001**	−0.01 (0.01)	0.31	−0.03 (0.01)	**<0.001**	−0.02 (0.01)	**0.012**	−0.06 (0.01)	**<0.001**
ACE	−0.01 (0.00)	**<0.001**	−0.02 (0.00)	**<0.001**	−0.03 (0.01)	**0.003**	−0.05 (0.01)	**<0.001**	−0.05 (0.01)	**<0.001**	−0.09 (0.01)	**<0.001**

PPC, parental psychological control; SI, social interactions; ACE, Adverse Childhood Experiences.

Corrected models include all variables in that column simultaneously as predictors; uncorrected models include age, sex, and each risk/protective factor separately as predictor.

*Note*: *p* values in bold indicate a significant effect following Holm's multiple comparison correction with initial *α* = 0.05; *p* values in italics indicate a significant effect after *α* = 0.05.

Since each column with uncorrected associations represents four separate models with different values for intercept, age, and sex, those values are not included in this table (see full model output online for coefficients, osf.io/6h7be/).

### Social processes and psychopathology

The four measures of psychopathology were all significantly associated with the three types of social processes (Table 5, uncorrected models). Higher levels of general psychopathology, anxiety, depression, and psychoticism were all associated with fewer interpersonal skills, and a lower quantity and quality of social interactions. When depression, anxiety, and psychoticism symptoms were entered simultaneously in the model, we only observed significant, unique associations between depressive symptoms and each of the three social processes (Table 5, corrected models).

**Table 5.** Associations between psychopathology and social processes

	Outcomes											
	Interpersonal skills				Quantity of SI				Quality of SI			
	Corrected		Uncorrected		Corrected		Uncorrected		Corrected		Uncorrected	
Predictors	*β* (s.e.)	*p*	*β* (s.e.)	*p*	*β* (s.e.)	*p*	*β* (s.e.)	*p*	*β* (s.e.)	*p*	*β* (s.e.)	*p*
Intercept	3.79 (0.02)	**<0.001**			0.98 (0.04)	**<0.001**			5.69 (0.04)	**<0.001**		
Age	−0.02 (0.01)	**0.003**	–		−0.04 (0.01)	**0.004**	–		−0.04 (0.01)	**0.001**	–	
Sex (ref = male)	0.10 (0.02)	**<0.001**	–		0.11 (0.05)	0.*043*	–		0.13 (0.05)	**0.003**	–	
Depressiveness	−0.14 (0.02)	**<0.001**	−0.14 (0.01)	**<0.001**	−0.17 (0.06)	**0.003**	−0.25 (0.03)	**<0.001**	−0.41 (0.05)	**0.001**	−0.47 (0.03)	**<0.001**
Anxiety	0.03 (0.02)	0.17	−0.09 (0.01)	**<0.001**	0.02 (0.05)	0.72	−0.19 (0.04)	**<0.001**	0.00 (0.05)	0.95	−0.36 (0.04)	**<0.001**
Psychoticism	−0.02 (0.03)	0.45	−0.14 (0.02)	**<0.001**	−0.12 (0.07)	0.07	−0.28 (0.04)	**<0.001**	−0.10 (0.06)	0.11	−0.49 (0.04)	**<0.001**
GSI	–		−0.18 (0.02)	**<0.001**		–	−0.34 (0.04)	**<0.001**		–	−0.62 (0.04)	**<0.001**

SI, social interactions; GSI, General Severity Index (general psychopathology score).

Corrected models include all variables (except for GSI) in that column simultaneously as predictors; uncorrected models include age, sex, and each type of psychopathology separately as predictor.

*Note*: *p* values in bold indicate a significant effect following Holm's multiple comparison correction with initial *α* = 0.05; *p* values in italics indicate a significant effect after *α* = 0.05.

Since each column with uncorrected associations represents four separate models with different values for intercept, age, and sex, those values are not included in this table (see full model output online for coefficients, osf.io/6h7be/).

### Age-related differences in effects

All differences in effects across age groups are represented by the interaction effects in online Supplementary Tables S1–S12 in the online Supplementary Material S5. Most of these interaction effects are not statistically significant, indicating that most investigated associations did not differ as a function of age. However, there are some statistically significant interaction effects (following multiple comparisons correction): The negative relationship between general psychopathology/depression/psychoticism and interpersonal skills is stronger for those in the 3rd or 5th grade, compared to those in the 1st grade (online Supplementary Tables S9/S10/S12; online Supplementary Material S5); the negative relationship between anxiety and interpersonal skills is stronger for those in the 5th grade than for those in the 1st grade (online Supplementary Table S11; online Supplementary Material S5); the negative relationship between general psychopathology/depression/psychoticism and the quality of daily-life social interactions is stronger for those in the 5th grade, compared to for those in the 1st grade (online Supplementary Tables S9/S10/S12; online Supplementary Material S5). Following the registered analysis plan on age-related effects, sensitivity analyses were conducted that excluded people whose age strongly differed from their classmates (i.e. at least two years younger or older than the mean age in their grade). These analyses showing no substantive difference with the main results (online Supplementary Material S1, osf.io/6h7be/).

## Discussion

The main aim of this study was to investigate the associations between known risk and protective factors, social processes, and general psychopathology in a general population sample of Flemish adolescents. We found significant associations between all types of variables: The social processes that we investigated were all meaningfully related to both risk/protective factors, and to different types of psychopathology. Although the conceptual model of a social mediation toward psychopathology still needs to be confirmed with longitudinal data (Fig. 1), these findings support the broad link between (day-to-day) social processes in youth mental health.

The reported results on the moderate prevalence of mental health symptoms are in line with previous research (Merikangas et al., 2010). Also, older adolescents reported more depressive symptoms than younger adolescents, and girls reported more symptoms (of every type) than boys. These findings are in line with other epidemiological work showing more depressive/anxiety psychopathology problems for girls than for boys (Campbell et al., 2021; Zahn-Waxler, Shirtcliff, & Marceau, 2008), and increased psychopathology as adolescents age (Kessler et al., 2007; Solmi et al., 2021).

As hypothesized, we consistently found the risk factors parental psychological control, bullying, and adverse childhood experiences to be associated with higher psychopathology levels, whereas more social support was associated with less psychopathology. This is in line with previous meta-analytic evidence highlighting how higher levels of parental psychological control (Pinquart, 2017), childhood trauma (McKay et al., 2021), and bullying (Moore et al., 2017), and lower levels of social support (Scardera et al., 2020), are all related to more mental health problems in adolescents and/or young adults.

However, the main added value of the proposed model and its tested associations, is that we also found unique relationships between these risk/protective factors and different *social processes* – both in terms of interpersonal skills and differential quantity/quality of daily social interactions. More adverse childhood experiences and psychological control were associated with fewer interpersonal skills and a lower quantity and quality of daily-life social interaction, while social support showed reverse associations. Also, we found higher levels of different types of psychopathology to be associated with fewer interpersonal skills and a lower quantity and quality of social interactions. This demonstrates that each of these factors, that we know to be related to mental health, and mental health itself, can be uniquely linked to how young people interact with each other.

Still, with these significant findings, we need to acknowledge that we cannot speak to the processes that underlie the reported associations. Given previous longitudinal evidence of the predictive effects of good social relations on mental health (Milner, Krnjacki, & LaMontagne, 2016), and the temporal precedence of most investigated risk/protective factors, we consider it likely that social processes (at least, to some extent) mediate the relationship between those risk/protective factors and psychopathology (cf. Fig. 1). However, given the cross-sectional and observational nature of the presented data, the veracity of the proposed mediation model remains to be tested.

It is also likely, for example, that latent psychopathology levels form the main driver of decreased interpersonal skills or lower levels of quantity/quality of social interactions (cf. the traditional conceptualization of social functioning as an outcome of mental health problems; e.g. Burns & Patrick, 2007). Learning more about the ordering of these effects is essential to better understand whether alterations in social processes are drivers, manifestations, or correlations of psychopathology. To inform on this temporal ordering of these effects (a necessary condition for determining the causality of effects; Shrout, 2011), longitudinal follow-up of these data is needed.

In addition, following leading theories of developmental psychopathology (Cicchetti & Toth, 1997), the underlying processes may also be inherently *transactional* in nature. Transactional models of development emphasize how maladaptation (here, psychopathology) can be produced by a dynamic, reciprocal interplay between the individual and (social) context (Sameroff & MacKenzie, 2003). This dynamic unfolding toward a range of mental health outcomes can be kick-started by a range of risk factors such as those investigated in this study. Transactional models of developmental psychopathology imply that it is in immediate feedback loops between individuals themselves and their proximal (social) environments that mental health develops. For future work, it would be valuable to test such models more directly, by assessing those transactions between individual and social context (e.g. with ESM), and ascertaining to what extent they predict longer-term development. Although all data collected here were cross-sectional in nature, the simultaneous inclusion of different age cohorts allows for a preliminary investigation of developmental differences. Notably, we observed strengthened associations between social processes and different types of psychopathology for older (as compared to younger) participants. The stronger relationship between (a lack of) interpersonal skills and psychopathology may reflect the developmental goal of adolescents to increasingly seek out others to connect with (Wrzus et al., 2013). As adolescents age, they generally engage in more social interactions with peers. Those with a lack of interpersonal skills may have lower-quality social interactions, which, in turn, may be driving the rise of psychopathology symptoms. This hypothesis is also supported by the strengthened association between the quality of social interactions and psychopathology symptoms for the oldest cohort in the current study.

This increased relevance of social factors – particularly in daily life – as adolescents age also provides a potential fruitful avenue for prevention and early intervention efforts. The key imperative to strengthen youth mental health efforts (McGorry, Purcell, Hickie, & Jorm, 2007) can be improved by focusing on those areas that are both meaningfully related to mental health and that are amenable to change. Although this study is unable to speak to the causal role of social processes in psychopathology development, previous evidence does strongly suggest the predictive adaptive effects of better social relations (Holt-Lunstad, Smith, & Layton, 2010; Milner et al., 2016). What requires further investigation, however, is the predictive effect of day-to-day social interaction (quality) in later mental health. If micro-level social processes (as investigated here) are indeed evidenced to be early warning signals if later psychopathological development, real-time interventions aimed at young people's daily social lives (such as Moderated Online Social Therapy+; Rice et al., 2018) may prove to be particularly effective. The moderate overlap between the different social processes investigated in this study suggests that they may each have their own unique value in this endeavor. What also needs to be considered in such developments, is the unique experience and potential benefit of *online* in addition to face-to-face interactions (Achterhof et al., 2022b) – and to what extent online social interactions are able to fulfil young people's social needs.

The results of this study should, however, be viewed in light of its limitations. First, since the data were cross-sectional, follow-up waves are needed to test the direction of effects. Second, due to the time constraint when collecting data, there is a relatively high level of missingness within the data – a limitation that was partially tackled by employing a state-of-the-art multiple imputation technique to handle missing data (Chhabra, Vashisht, & Ranjan, 2017). Within the ESM protocol, as well, participants in this study were less compliant than in similar ESM studies in adolescents (40% *v.* average of 74%, van Roekel, Keijsers, & Chung, 2019), potentially leading to biased results. Despite this low compliance, the relatively high number of daily prompts (10) still gives us a large amount of data to draw on, while the short response rate to each questionnaire (90 s) and the prompting in naturalistic school settings are unique features ensuring high ecological validity of the data. Third, our bullying measure only consisted of two items on physical bullying, thereby not capturing the full scope of bullying (excluding e.g. emotional, verbal, and cyberbullying).

Two additional strengths should be noted. This study is one of the largest experience-sampling datasets in existence, thereby providing novel insights into the relationships between adolescents' day-to-day social lives and their mental health. In addition, the adherence to open-science practices (registration of the analyses pre-data analysis, the online publication of code and materials) strengthens the robustness, replicability, and reproducibility of the reported findings.

## Conclusion

In this study, we confirmed that, in adolescents from the general population, social processes (social skills, quantity, and quality of everyday social interactions) are associated with risk and protective factors for psychopathology, and with depression, anxiety, and psychotic symptoms. Although further longitudinal work is required to verify the directionality of these associations, these results highlight the transdiagnostic relevance of social processes for different types of early psychopathology – perhaps even more so for late adolescence. A focus on everyday social interactions may aid in a better understanding of how known risk/protective factors can lead to different types of psychopathology in young people. Moreover, given the importance and targetable nature of social processes, an increased social focus will prove valuable in boosting prevention and intervention efforts.

## Supporting information

Achterhof et al. supplementary material Achterhof et al. supplementary material

## Supplementary material

The supplementary material for this article can be found at https://doi.org/10.1017/S0033291724001594
