# Supplementary material for: Social processes as the missing link: cross-sectionally testing a conceptual model on social mediators of early psychopathological development
Source: Psychol Med. 2024 Oct 23;54(13):3591–601. doi: 10.1017/S0033291724001594 (PMC11536120; doi:10.1017/S0033291724001594)
Supplement: Achterhof et al. supplementary material [file S0033291724001594sup001.docx]

# Online Supplementary Materials for “Environmental, inter- and intrapersonal risk factors and their association with psychopathology symptoms among adolescents: Cross-sectional results from the SIGMA study”

# Supplement 1: Sensitivity Analyses

## Analyses with trauma score based on full list of items

In the analyses described in the manuscript, we used an adapted total trauma score of the Juvenile Victimization Questionnaire (JVQ; Hamby et al., 2004) that excluded the Conventional Crime module. This was done because this module was not presented to all first-grade students. As described in the registration for this study, we also performed a set of sensitivity analyses of all associations involving the trauma variable – now defined by the trauma score as based on the full list of JVQ items. The results of these sensitivity analyses are presented in the two tables below.

*Table 1: Associations between risk/protective factors and psychopathology*

|  | **Outcomes** | | | | | | | | | | | | | | | | |
| --- | --- | --- | --- | --- | --- | --- | --- | --- | --- | --- | --- | --- | --- | --- | --- | --- | --- |
|  | **GSI** | | | | **Depression** | | | | **Anxiety** | | | | **Psychoticism** | | | | |
|  | **Corrected** | | **Uncorrected** | | **Corrected** | | **Uncorrected** | | **Corrected** | | **Uncorrected** | | **Corrected** | | **Uncorrected** | | |
| **Predictors** | *β (SE)* | *p* | *β (SE)* | *p* | *β (SE)* | *p* | *β (SE)* | *p* | *β (SE)* | *p* | *β (SE)* | *p* | *β (SE)* | *p* | *β (SE)* | *p* |  |
| Intercept | .73 (.02) | **<.001** | -.16 (.02) | **<.001** | .61 (.03) | **<.001** | -.20 (.03) | **<.001** | .75 (.02) | **<.001** | .74 (.03) | **<.001** | .56 (.02) | **<.001** | .54 (.02) | **<.001** |  |
| Age | .02 (.01) | **.005** | .03 (.01) | **<.001** | .05 (.01) | **<.001** | .07 (.01) | **<.001** | .00 (.01) | .58 | .02 (.01) | .059 | .02 (.01) | *.024* | .04 (.01) | **<.001** |  |
| Sex (ref = male) | .23 (.02) | **<.001** | .25 (.03) | **<.001** | .30 (.03) | **<.001** | .32 (.04) | **<.001** | .22 (.03) | **<.001** | .25 (.03) | **<.001** | .18 (.03) | **<.001** | .20 (.03) | **<.001** |  |
| PPC | .23 (.02) | **<.001** |  |  | .24 (.03) | **<.001** |  |  | .20 (.03) | **<.001** |  |  | .23 (.03) | **<.001** |  |  |  |
| Social support | -.09 (.02) | **<.001** |  |  | -.23 (.03) | **<.001** |  |  | -.01 (.03) | .77 |  |  | -.13 (.03) | **<.001** |  |  |  |
| Bullying | .03 (.00) | **<.001** |  |  | .05 (.01) | **<.001** |  |  | .03 (.01) | **<.001** |  |  | .03 (.01) | **<.001** |  |  |  |
| **Full trauma score** | .04 (.00) | **<.001** | .06 (.00) | **<.001** | .04 (.00) | **<.001** | .07 (.04) | **<.001** | .04 (.00) | **<.001** | .06 (.00) | **<.001** | .04 (.00) | **<.001** | .06 (.00) | **<.001** |  |

*Note: p-values in bold indicate a significant effect following Holm’s multiple comparison correction with initial α=.05; p-values in italics indicate a significant effect after α=.05*

*PPC = parental psychological control; GSI = General Severity Index (general psychopathology)*

*Table 2: Associations between risk/protective factors and social processes*

|  |  |  | |  | | |  | | **Outcomes** | | | | |  | | |  | |
| --- | --- | --- | --- | --- | --- | --- | --- | --- | --- | --- | --- | --- | --- | --- | --- | --- | --- | --- |
|  | **Interpersonal skills** | | | | | | | **Quantity of SI** | | | | | **Quality of SI** | | | | | |
|  | **Corrected** | | | | **Uncorrected** | | | **Corrected** | | | **Uncorrected** | | **Corrected** | | | **Uncorrected** | | |
| **Predictors** | *β (SE)* | | *p* | | *β (SE)* | *p* | | *β (SE)* | | *p* | *β (SE)* | *p* | *β (SE)* | | *p* | *β (SE)* | | *p* |
| Intercept | 3.84 (.01) | | **<.001** | | 3.82 (.02) | **<.001** | | -1.03 (.04) | | **<.001** | -1.03 (.04) | <.001 | 5.80 (.03) | | **<.001** | 5.79 (.04) | | **<.001** |
| Age | -.01 (.00) | | **.008** | | -.02 (.01) | **<.001** | | .04 (.01) | | **.004** | .05 (.01) | <.001 | -.03 (.01) | | *.010* | -.06 (.01) | | **<.001** |
| Sex (ref = male) | .02 (.02) | | .30 | | .05 (.02) | **.011** | | -.02 (.05) | | .75 | -.02 (.05) | .67 | -.04 (.04) | | .32 | -.03 (.05) | | .58 |
| PPC | -.04 (.02) | | **.007** | |  |  | | .12 (.05) | | **.013** |  |  | -.29 (.04) | | **<.001** |  | |  |
| Social support | .43 (.02) | | **<.001** | |  |  | | -.21 (.05) | | **<.001** |  |  | .55 (.04) | | **<.001** |  | |  |
| Bullying | .00 (.00) | | .20 | |  |  | | .01 (.01) | | .44 |  |  | -.02 (.01) | | *.046* |  | |  |
| **Full trauma score** | -.01 (.00) | | **<.001** | | -.02 (.00) | **<.001** | | .23 (.01) | | **<.001** | .03 (.01) | **<.001** | -.03 (.01) | | **<.001** | -.06 (.01) | | **<.001** |

*Note: p-values in bold indicate a significant effect following Holm’s multiple comparison correction with initial α=.05; p-values in italics indicate a significant effect after α=.05*

*PPC = parental psychological control; SI = social interactions*

## Analyses with participants outside of age range excluded

Note that we also performed another set of sensitivity analyses for the analyses pertaining to the moderating effect of school cohort on all analyses, following the rationale that was described in the study’s registration (osf.io/jhav7): *“Because in these exploratory analyses, each cohort is defined by the class grade (1st, 3rd, or 5th grade of secondary education) rather than by age, participants who are older or younger than their classmates might affect these results. Therefore, we will also conduct a series of sensitivity analyses for all analyses of RQ5, where all participants are removed who are not within two years of the mean age in their cohort.”*

It turned out that 20 participants in the full sample fell outside the age range of their cohort, and were thus excluded in these sensitivity analyses. Excluding these participants did not substantively alter any of the results of these research questions. Given the large amount of results that only slightly differ from those presented in Supplement 3, the results of these sensitivity analyses are not presented here, but are available online ([osf.io/6h7be/](https://osf.io/6h7be/)).

# Supplement 2: ESM questionnaire

The SIGMA ESM questionnaire is also publicly available in the ESM Item Repository (www.esmitemrepository.com; Kirtley et al., 2020; dataset: ‘sigma’).

If not multiple choice (MC), items will be rated on a 7-point Likert scale, ranging from 1 (not at all) to 7 (very much).

## Beep questionnaire throughout the day

### Positive and negative affect

1) Ik voel me vrolijk (I feel cheerful)

2) Ik voel me geïrriteerd (I feel irritated)

3) Ik voel me angstig (I feel anxious)

4) Ik voel me tevreden (I feel satisfied)

5) Ik voel me onzeker (I feel insecure)

6) Ik voel me eenzaam (I feel lonely)

7) Ik voel me ontspannen (I feel relaxed)

8) Ik voel me wantrouwig (I feel paranoid)

9) Ik voel me verdrietig (I feel sad)

10) Ik voel me gestresst (I feel stressed)

11) Ik voel me rusteloos (I feel restless)

12) Over het algemeen gezien voel ik me nu goed (In general I feel well at the moment)

13) Ik kan me goed concentreren (I can concentrate well)

14) Ik voel me zeker van mezelf (How much self-confidence do you have?)

### Identity

15) Ik kan mezelf zijn (I can be myself)

16) Ik twijfel wie ik ben (I doubt who I am)

17) Ik weet waar ik voor sta (I know where I stand for)

### Social context

18) Met wie ben ik? (Who is with me?)

MC: Familie thuis (gezin) - andere familie (other family) - vriend(en) (friends) – andere leeftijdsgenoten (other peers) - leraar (teacher) - onbekende mensen (unknown people/others) - niemand (nobody)

*If alone:*

19) Ik vind het fijn om alleen te zijn (I find being alone pleasant)

20) Ik wil alleen zijn (I want to be alone)

21) Ik voel me buitengesloten (I feel an outsider)

22) Ik wil liever met anderen zijn (I prefer to have company)

*If in company:*

19) We zijn samen iets aan het doen (praten, leren, gamen, etc.) (We are doing something together (talking, learning, gaming, etc.))

20) Ik voel me op mijn gemak in dit gezelschap (I feel comfortable in this company)

21) Ik voel me gewaardeerd door dit gezelschap (I feel valued in this company)

22) Ik hoor erbij

23) Ik zou liever alleen zijn (I prefer to be alone)

24) Ik ben virtueel in contact met anderen (I’m interacting with others online)

MC: Ja – Nee

*If yes:*

25) We zijn samen iets aan het doen (We’re interacting)

26) Ik voel me op mijn gemak in dit gezelschap (I feel comfortable in this company)

27) Ik voel me gewaardeerd door dit gezelschap (I feel valued in this company)

28) Ik hoor erbij (I belong to the people I’m interacting with)

*If no:*

25) Ik zou graag virtueel in contact zijn met anderen (I would like to be contact with others, virtually)

26) Hier heb ik zelf voor gekozen (This is my choice)

27) Ik voel me buitengesloten (I feel an outsider)

### Physical context

29) Wat ben je aan het doen? (What are you doing?)

MC: vrije tijd actief (sporten, uitgaan) (leisure active) – vrije tijd passief (tv kijken, lezen) (leisure passive) – school/werk (school/job) – huishoudelijke taken (chores) - onderweg (on my way) - persoonlijke hygiene (wassen/tanden poetsen/aankleden) (personal hygiene) – iets anders (other) - niets (nothing)

30) Dit vind ik leuk (I like this activity)

31) Ik zou liever iets anders doen (I would rather do something else)

32) Dit is moeilijk voor mij (This is difficult for me)

33) Ik kan dit goed (I can do this well)

34) Ik ben actief bezig (I’m engaged)

35) Waar ben je? (Where are you?)

MC: op school (at school) - thuis (at home) - bij vriend(en) (at friends’ place) - bij andere familie dan thuis (other family than home) - op de fiets/in de auto/in de bus/in de trein (public transport) - ergens anders binnen (other indoors) – ergens anders buiten (other outdoors)

### Control items

36) Ik ben moe (I am tired)

37) Ik heb honger (I am hungry)

38) Ik voel me niet lekker (I feel physically unwell)

39) Ik heb pijn (I am in pain)

### Anticipation of pleasure

Denk aan de belangrijkste situatie voor jou in de komende 2 uur (Think about the most important situation for you in the next 2 hours)

40) Hoeveel zin heb je in deze situatie? (How much are you looking forward to this situation?)

41) Deze situatie hoort bij de categorie: (This situation belongs tot he following category:)

MC: lichamelijke inspanning (physical activity) – school/werk (school/job) – actieve ontspanning (active relaxation) – passieve ontspanning (passive relaxation) – slapen (sleeping) – iets anders (something else) – eten/drinken (eating/drinking) – niets (nothing)

### Suicidal ideation/self-harm

Sinds de vorige beep: (Since the last beep:)

42) Heb je eraan gedacht om jezelf te verwonden? (Have you considered harming yourself?)

*If rated with 2-7:**

43) In welke mate had je tijdens deze gedachten over zelfverwonding de wens om je leven te

beëindigen? (During these self-harm thoughts, how much did you wish to end your life?)

44) Heb je hiernaar gehandeld door jezelf opzettelijk te verwonden, pijn te doen of te vergiftigen?

(Have you actually harmed yourself on purpose?)

MC: Ja – Nee (Yes – No)

### Substance use

45) Sinds de vorige beep heb ik het volgende gebruikt: (Since the last beep I’ve used:)

MC: niets (nothing); medicatie (medication); alcohol (alcohol); sigaretten (cigarettes);

cannabis (cannabis); andere drugs (other drugs)

46) Deze beep stoorde me (This beep disturbed me)

*** Pop-up will show if participants report thoughts on suicide and/or self-harm:**

Het lijkt erop dat je het op dit moment misschien moeilijk hebt. Onthoud dat als je anoniem met iemand wilt praten over hoe je je voelt, dat kan op:

Awel op 102, tussen 16u - 22u; Tele-Onthaal op 106, 24 uur per dag; Zelfmoordlijn, 1813, 24 uur per dag.

Het kan natuurlijk ook altijd goed zijn om met iemand te praten die dicht bij je staat, zoals een vriend, ouder, of leraar.

Mocht er een noodgeval zijn, en je hebt direct medische hulp nodig, bel dan gelijk 112.

**English translation of pop-up:**

It looks like you’re having a difficult time at the moment. Remember that if you would like to talk to someone anonymously about how you are feeling, you can: AWEL on 102, between 16:00 – 22:00; Tele-Onthaal on 106, 24 hours a day; the Flemish suicide crisis line 1813, 24 hours a day. Of course, it’s always good if you can talk to someone close to you, like a friend, parent or teacher. If you are in an emergency situation and you need immediate medical help, call 112.

# Supplement 3: Deviations from post-registration

In the post-registration, we originally described to use the total sum score of the parental psychological control variable. However, to enhance interpretability of the regression coefficients related to this variable, we decided to use the total *mean* score of this variable.

In the multilevel models described in the post-registration, we erroneously omitted to include an autoregressive structure, which we added to our final models.

# Supplement 4: Descriptives of raw data

The descriptive information of the included variables in Table 1 in the manuscript is based on pooled estimates of the full multiple-imputed datasets. Here, we present the descriptive information of all variables without dealing with the missing data, and the total available sample available for each variable. Note that the separate descriptives of neither age group is listed here (as there was no missing data for this variable), nor of the mean quantity/quality of social interactions (as these moment-level variables had not been multiply imputed).

*Table 1: Descriptive characteristics of full sample, pooled across the 20 multiply imputed datasets*

| **Variables** | **Full sample (n = 1913)** | | | |
| --- | --- | --- | --- | --- |
|  | *Available n* | *Mean (SD)* | *Median* | *Range* |
| Age | 1907 | 13.8 (1.9) | 13.0 | 11.0 – 20.0 |
| Gender (%girls) | 1902 | 69.6 |  |  |
| Parental psychological control | 1329 | 1.9 (.6) | 1.8 | 1.0 – 4.4 |
| Social support | 1657 | 1.9 (.5) | 1.9 | 0.0 – 3.0 |
| Bullying | 1368 | 1.8 (3.1) | 1.0 | 0.0 – 12.0 |
| Trauma | 1226 | 3.8 (3.3) | 3.0 | 0.0 – 24.0 |
| Interpersonal skills | 1580 | 3.9 (4.3) | 3.9 | 1.0 – 5.0 |
| Depression | 1501 | 0.8 (0.9) | 0.5 | 0.0 – 4.0 |
| Anxiety | 1542 | 0.9 (0.7) | 0.7 | 0.0 – 4.0 |
| Psychoticism | 1521 | 0.7 (0.7) | 0.4 | 0.0 – 3.2 |
| Psychopathology – total (GSI) | 1346 | 0.9 (0.6) | 0.7 | 0.0 – 3.4 |

# Supplement 5: Results including interaction effects between each predictor and grade

*Table 1: Linear regression results, predicting different types of psychopathology from parental psychological control (PPC), school grade, and the interaction between grade and PPC.*

| **Outcomes** | | | | | | | | |
| --- | --- | --- | --- | --- | --- | --- | --- | --- |
|  | **GSI** | | **Depression** | | **Anxiety** | | **Psychoticism** | |
| **Predictors** | *β (SE)* | *p* | *β (SE)* | *p* | *β (SE)* | *p* | *β (SE)* | *p* |
| Intercept | .70 (.02) | **<.001** | .53 (.03) | **<.001** | .74 (.03) | **<.001** | .50 (.03) | **<.001** |
| Sex (ref = male) | .20 (.03) | **<.001** | .27 (.04) | **<.001** | .21 (.03) | **<.001** | .16 (.03) | **<.001** |
| PPC | .41 (.03) | **<.001** | .43 (.04) | **<.001** | .41 (.04) | **<.001** | .38 (.04) | **<.001** |
| Grade 3 (ref = grade 1) | .07 (03) | *.033* | .12 (.05) | **.009** | .00 (.04) | .91 | .12 (.04) | **.003** |
| Grade 5 (ref = grade 1) | .15 (.03) | **<.001** | .29 (.04) | **<.001** | .11 (.04) | **.007** | .17 (.04) | **<.001** |
| Grade 3*PPC | -.02 (.05) | .76 | .01 (.07) | .88 | -.06 (.06) | .33 | .02 (.06) | .76 |
| Grade 5*PPC | .01 (.05) | .90 | .09 (.07) | .22 | -.07 (.06) | .23 | .06 (.06) | .33 |

*Note: p-values in bold indicate a significant effect following Holm’s multiple comparison correction with initial α=.05; p-values in italics indicate a significant effect after α=.05*

*PPC = parental psychological control; GSI = General Severity Index (general psychopathology)*

*Table 2: Linear regression results, predicting different types of psychopathology from social support, school grade, and the interaction between grade and social support.*

| **Outcomes** | | | | | | | | |
| --- | --- | --- | --- | --- | --- | --- | --- | --- |
|  | **GSI** | | **Depression** | | **Anxiety** | | **Psychoticism** | |
| **Predictors** | *β (SE)* | *p* | *β (SE)* | *p* | *β (SE)* | *p* | *β (SE)* | *p* |
| Intercept | .64 (.03) | **<.001** | .46 (.03) | **<.001** | .69 (.03) | **<.001** | .45 (.03) | **<.001** |
| Sex (ref = male) | .23 (.03) | **<.001** | .31 (.04) | **<.001** | .23 (.03) | **<.001** | .19 (.03) | **<.001** |
| Social support | -.23 (.04) | **<.001** | -.35 (.05) | **<.001** | -.17 (.05) | **<.001** | -.25 (.04) | **<.001** |
| Grade 3 (ref = grade 1) | .16 (.04) | **<.001** | .20 (.05) | **<.001** | .09 (.04) | *.041* | .20 (.04) | **<.001** |
| Grade 5 (ref = grade 1) | .26 (.03) | **<.001** | .40 (.04) | **<.001** | .21 (.04) | **<.001** | .27 (.04) | **<.001** |
| Grade 3*Social support | -.01 (.07) | .89 | -.06 (.09) | .50 | .01 (.08) | .88 | -.03 (.08) | .73 |
| Grade 5*Social support | .03 (.07) | .62 | -.11 (.09) | .25 | .16 (.09) | *.049* | -.01 (.08) | .90 |

*Note: p-values in bold indicate a significant effect following Holm’s multiple comparison correction with initial α=.05; p-values in italics indicate a significant effect after α=.05*

*GSI = General Severity Index (general psychopathology)*

*Table 3: Linear regression results, predicting different types of psychopathology from bullying, school grade, and the interaction between grade and bullying.*

| **Outcomes** | | | | | | | | |
| --- | --- | --- | --- | --- | --- | --- | --- | --- |
|  | **GSI** | | **Depression** | | **Anxiety** | | **Psychoticism** | |
| **Predictors** | *β (SE)* | *p* | *β (SE)* | *p* | *β (SE)* | *p* | *β (SE)* | *p* |
| Intercept | .67 (.02) | **<.001** | .51 (.03) | **<.001** | .72 (.03) | **<.001** | .48 (.03) | **<.001** |
| Sex (ref = male) | .19 (.03) | **<.001** | .24 (.04) | **<.001** | .19 (.03) | **<.001** | .14 (.03) | **<.001** |
| Bullying | .06 (.01) | **<.001** | .08 (.01) | **<.001** | .06 (.01) | **<.001** | .06 (.01) | **<.001** |
| Grade 3 (ref = grade 1) | .15 (.03) | **<.001** | .20 (.04) | **<.001** | .08 (.04) | .053 | .20 (04) | **<.001** |
| Grade 5 (ref = grade 1) | .22 (.03) | **<.001** | .36 (.04) | **<.001** | .17 (.04) | **<.001** | .24 (.04) | **<.001** |
| Grade 3*Bullying | .01 (.01) | .68 | .02 (.02) | .29 | .01 (.01) | .70 | .01 (.02) | .42 |
| Grade 5*Bullying | -.00 (.01) | .94 | .00 (.01) | .71 | -.00 (.01) | .81 | -.00 (.01) | .83 |

*Note: p-values in bold indicate a significant effect following Holm’s multiple comparison correction with initial α=.05; p-values in italics indicate a significant effect after α=.05*

*GSI = General Severity Index (general psychopathology)*

*Table 4: Linear regression results, predicting different types of psychopathology from trauma, school grade, and the interaction between grade and trauma.*

| **Outcomes** | | | | | | | | |
| --- | --- | --- | --- | --- | --- | --- | --- | --- |
|  | **GSI** | | **Depression** | | **Anxiety** | | **Psychoticism** | |
| **Predictors** | *β (SE)* | *p* | *β (SE)* | *p* | *β (SE)* | *p* | *β (SE)* | *p* |
| Intercept | .70 (.02) | **<.001** | .53 (.03) | **<.001** | .74 (.03) | **<.001** | .51 (.03) | **<.001** |
| Sex (ref = male) | .21 (.03) | **<.001** | .28 (.04) | **<.001** | .21 (.03) | **<.001** | .17 (.03) | **<.001** |
| Trauma | .09 (.01) | **<.001** | .10 (.01) | **<.001** | .08 (.01) | **<.001** | .09 (.01) | **<.001** |
| Grade 3 (ref = grade 1) | .09 (.03) | **.008** | .13 (.04) | **.002** | .02 (.04) | .60 | .13 (.04) | **<.001** |
| Grade 5 (ref = grade 1) | .11 (.03) | **<.001** | .26 (.04) | **<.001** | .06 (.04) | .11 | .14 (.04) | **<.001** |
| Grade 3*Trauma | -.01 (.01) | .21 | -.01 (.01) | .54 | -.01 (.01) | .55 | -.01 (.01) | .31 |
| Grade 5*Trauma | -.01 (.01) | .47 | -.00 (.01) | .73 | .01 (.01) | .63 | -,01 (.01) | .37 |

*Note: p-values in bold indicate a significant effect following Holm’s multiple comparison correction with initial α=.05; p-values in italics indicate a significant effect after α=.05*

*GSI = General Severity Index (general psychopathology)*

*Table 5: Linear regression results, predicting different types of social processes from parental psychological control (PPC), school grade, and the interaction between grade and PPC.*

|  | **Outcomes** | | | | | |
| --- | --- | --- | --- | --- | --- | --- |
|  | **Interpersonal functioning** | | **Quantity of SI** | | **Quality of SI** | |
| **Predictors** | *β (SE)* | *p* | *β (SE)* | *p* | *β (SE)* | *p* |
| Intercept | 3.85 (.02) | **<.001** | -1.08 (.05) | **<.001** | 5.83 (.04) | **<.001** |
| Sex (ref = male) | .07 (.02) | **.002** | -.05 (.05) | .33 | .04 (.05) | .44 |
| PPC | -.15 (.02) | **<.001** | .24 (.06) | **<.001** | -.50 (.05) | **<.001** |
| Grade 3 (ref = grade 1) | -.10 (03) | **<.001** | .06 (.07) | .34 | -.21 (.06) | **<.001** |
| Grade 5 (ref = grade 1) | -.09 (.03) | **<.001** | .23 (.06) | **<.001** | -.14 (.06) | **.014** |
| Grade 3*PPC | -.03 (.04) | .45 | .04 (.11) | .70 | -.10 (.09) | .31 |
| Grade 5*PPC | .09 (.04) | *.028* | -.02 (.10) | .81 | .02 (.09) | .86 |

*Note: p-values in bold indicate a significant effect following Holm’s multiple comparison correction with initial α=.05; p-values in italics indicate a significant effect after α=.05*

*PPC = parental psychological control; SI =social interactions*

*Table 6: Linear regression results, predicting different types of social processes from social support, school grade, and the interaction between grade and social support.*

|  | **Outcomes** | | | | | |
| --- | --- | --- | --- | --- | --- | --- |
|  | **Interpersonal functioning** | | **Quantity of SI** | | **Quality of SI** | |
| **Predictors** | *β (SE)* | *p* | *β (SE)* | *p* | *β (SE)* | *p* |
| Intercept | 3.87 (.02) | **<.001** | -1.12 (.05) | **<.001** | 5.92 (.04) | **<.001** |
| Sex (ref = male) | .03 (.02) | .17 | -.02 (.05) | .71 | -.03 (.05) | .45 |
| Social support | .48 (.02) | **<.001** | -.30 (.07) | **<.001** | .61 (.06) | **<.001** |
| Grade 3 (ref = grade 1) | -.09 (.02) | **<.001** | .10 (.06) | .10 | -.28 (.06) | **<.001** |
| Grade 5 (ref = grade 1) | -.08 (.02) | **<.001** | .27 (.06) | **<.001** | -.23 (.06) | **<.001** |
| Grade 3*Social support | -.05 (.04) | .21 | .03 (.13) | .79 | .23 (.11) | *.040* |
| Grade 5*Social support | -.08 (.04) | .058 | .06 (.13) | .62 | .05 (.11) | .66 |

*Note: p-values in bold indicate a significant effect following Holm’s multiple comparison correction with initial α=.05; p-values in italics indicate a significant effect after α=.05*

*SI =social interactions*

*Table 7: Linear regression results, predicting different types of social processes from bullying, school grade, and the interaction between grade and bullying.*

|  | **Outcomes** | | | | | |
| --- | --- | --- | --- | --- | --- | --- |
|  | **Interpersonal functioning** | | **Quantity of SI** | | **Quality of SI** | |
| **Predictors** | *β (SE)* | *p* | *β (SE)* | *p* | *β (SE)* | *p* |
| Intercept | 3.86 (.02) | **<.001** | -1.10 (.05) | **<.001** | 5.87 (.04) | **<.001** |
| Sex (ref = male) | .07 (.02) | **.002** | -.05 (.05) | .31 | .05 (.05) | .33 |
| Bullying | -.02 (.01) | **.001** | .04 (.01) | **.004** | -.05 (.01) | **<.001** |
| Grade 3 (ref = grade 1) | -.13 (.03) | **<.001** | .12 (.06) | .07 | -.32 (.06) | **<.001** |
| Grade 5 (ref = grade 1) | -.11 (.03) | **`<.001** | .28 (.06) | **<.001** | -.24 (.06) | **<.001** |
| Grade 3*Bullying | .01 (.01) | .20 | -.00 (.02) | .85 | -.04 (.02) | *.037* |
| Grade 5*Bullying | .01 (.01) | .12 | -.02 (.02) | .20 | .01 (.02) | .70 |

*Note: p-values in bold indicate a significant effect following Holm’s multiple comparison correction with initial α=.05; p-values in italics indicate a significant effect after α=.05*

*SI =social interactions*

*Table 8: Linear regression results, predicting different types of social processes from trauma, school grade, and the interaction between grade and trauma.*

|  | **Outcomes** | | | | | |
| --- | --- | --- | --- | --- | --- | --- |
|  | **Interpersonal functioning** | | **Quantity of SI** | | **Quality of SI** | |
| **Predictors** | *β (SE)* | *P* | *β (SE)* | *p* | *β (SE)* | *p* |
| Intercept | 3.85 (.02) | **<.001** | -1.08 (.05) | **<.001** | 5.84 (.04) | **<.001** |
| Sex (ref = male) | .06 (.02) | **.003** | -.04 (.05) | .46 | .02 (.04) | .66 |
| Trauma | -.02 (.00) | **<.001** | .06 (.01) | **<.001** | -.09 (.01) | **<.001** |
| Grade 3 (ref = grade 1) | -.11 (.03) | **<.001** | .07 (.06) | .28 | -.25 (.06) | **<.001** |
| Grade 5 (ref = grade 1) | -.09 (.03) | **<.001** | .23 (.07) | **<.001** | -.14 (.06) | *.017* |
| Grade 3*Trauma | -.01 (.01) | .19 | -.00 (.02) | .87 | -.02 (.02) | .23 |
| Grade 5*Trauma | .01 (.01) | .14 | -.03 (.02) | .15 | .01 (.02) | .55 |

*Note: p-values in bold indicate a significant effect following Holm’s multiple comparison correction with initial α=.05; p-values in italics indicate a significant effect after α=.05*

*SI =social interactions*

*Table 9: Linear regression results, predicting different types of social processes from general psychopathology, school grade, and the interaction between grade and general psychopathology.*

|  | **Outcomes** | | | | | |
| --- | --- | --- | --- | --- | --- | --- |
|  | **Interpersonal functioning** | | **Quantity of SI** | | **Quality of SI** | |
| **Predictors** | *β (SE)* | *P* | *β (SE)* | *p* | *β (SE)* | *p* |
| Intercept | 3.82 (.01) | **<.001** | -.1.04 (.05) | **<.001** | 5.76 (.04) | **<.001** |
| Sex (ref = male) | .10 (.01) | **<.001** | -.11 (.05) | *.042* | .14 (.05) | **.002** |
| GSI | -.25 (.01) | **<.001** | .38 (.06) | **<.001** | -.67 (.06) | **<.001** |
| Grade 3 (ref = grade 1) | -.06 (.01) | **<.001** | .07 (.06) | .30 | -.22 (.06) | **<.001** |
| Grade 5 (ref = grade 1) | -.03 (.01) | **.002** | .21 (.06) | **.001** | -.14 (.06) | **.014** |
| Grade 3*GSI | .07 (.02) | **<.001** | -.05 (.11) | .62 | -.06 (.09) | .49 |
| Grade 5*GSI | .11 (.01) | **<.001** | -.10 (.10) | .31 | .24 (.09) | **.009** |

*Note: p-values in bold indicate a significant effect following Holm’s multiple comparison correction with initial α=.05; p-values in italics indicate a significant effect after α=.05*

*SI =social interactions; GSI = General Severity Index (general psychopathology)*

*Table 10: Linear regression results, predicting different types of social processes from depressive symptoms, school grade, and the interaction between grade and depressive symptoms.*

|  | **Outcomes** | | | | | |
| --- | --- | --- | --- | --- | --- | --- |
|  | **Interpersonal functioning** | | **Quantity of SI** | | **Quality of SI** | |
| **Predictors** | *β (SE)* | *P* | *β (SE)* | *p* | *β (SE)* | *p* |
| Intercept | 3.82 (.01) | **<.001** | -1.04 (.05) | **<.001** | 5.75 (.04) | **<.001** |
| Sex (ref = male) | .10 (.01) | **<.001** | -.11 (.05) | *.045* | .14 (.05) | **.002** |
| Depression | -.21 (.01) | **<.001** | .30 (.05) | **<.001** | -.54 (.04) | **<.001** |
| Grade 3 (ref = grade 1) | -.06 (.01) | **<.001** | .07 (.06) | .27 | -.22 (.06) | **<.001** |
| Grade 5 (ref = grade 1) | -.02 (.01) | *.025* | .19 (.07) | **.004** | -.10 (.06) | .07 |
| Grade 3*Depression | .06 (.01) | **<.001** | -.07 (.08) | .37 | -.01 (.07) | .92 |
| Grade 5*Depression | .11 (.01) | **<.001** | -.09 (.07) | .25 | .20 (.07) | **.002** |

*Note: p-values in bold indicate a significant effect following Holm’s multiple comparison correction with initial α=.05; p-values in italics indicate a significant effect after α=.05*

*SI =social interactions*

*Table 11: Linear regression results, predicting different types of social processes from anxiety symptoms, school grade, and the interaction between grade anxiety symptoms.*

|  | **Outcomes** | | | | | |
| --- | --- | --- | --- | --- | --- | --- |
|  | **Interpersonal functioning** | | **Quantity of SI** | | **Quality of SI** | |
| **Predictors** | *β (SE)* | *P* | *β (SE)* | *p* | *β (SE)* | *p* |
| Intercept | 3.84 (.01) | **<.001** | -1.08 (.05) | **<.001** | 5.83 (.04) | **<.001** |
| Sex (ref = male) | .08 (.01) | **<.001** | -.08 (.05) | .13 | .09 (.05) | .06 |
| Anxiety | -.14 (.01) | **<.001** | .21 (.05) | **<.001** | -.39 (.05) | **<.001** |
| Grade 3 (ref = grade 1) | -.09 (.01) | **<.001** | .11 (.06) | .09 | -.31 (.06) | **<.001** |
| Grade 5 (ref = grade 1) | -.07 (.01) | **<.001** | .26 (.06) | **<.001** | -.22 (.06) | **<.001** |
| Grade 3*Anxiety | .03 (.02) | .06 | -.01 (.09) | .88 | -.05 (.08) | .53 |
| Grade 5*Anxiety | .10 (.01) | **<.001** | -.06 (.09) | .49 | .18 (.08) | *.034* |

*Note: p-values in bold indicate a significant effect following Holm’s multiple comparison correction with initial α=.05; p-values in italics indicate a significant effect after α=.05*

*SI =social interactions*

*Table 12: Linear regression results, predicting different types of social processes from psychotic symptoms, school grade, and the interaction between grade and psychotic symptoms.*

|  | **Outcomes** | | | | | |
| --- | --- | --- | --- | --- | --- | --- |
|  | **Interpersonal functioning** | | **Quantity of SI** | | **Quality of SI** | |
| **Predictors** | *β (SE)* | *P* | *β (SE)* | *p* | *β (SE)* | *p* |
| Intercept | 3.83 (.01) | **<.001** | -1.06 (.05) | **<.001** | 5.80 (.04) | **<.001** |
| Sex (ref = male) | .08 (.01) | **<.001** | -.08 (.05) | .11 | .09 (.05) | *.047* |
| Psychoticism | -.21 (.01) | **<.001** | .32 (.06) | **<.001** | -.54 (.05) | **<.001** |
| Grade 3 (ref = grade 1) | -.07 (.01) | **<.001** | .07 (.06) | .28 | -.23 (.06) | **<.001** |
| Grade 5 (ref = grade 1) | -.04 (.01) | **<.001** | .21 (.06) | **.001** | -.16 (.06) | **.005** |
| Grade 3*Psychoticism | .10 (.02) | **<.001** | -.09 (.10) | .35 | -.02 (.08) | .83 |
| Grade 5*Psychoticism | .10 (.01) | **<.001** | -.06 (.09) | .49 | .21 (.08) | **.011** |

*Note: p-values in bold indicate a significant effect following Holm’s multiple comparison correction with initial α=.05; p-values in italics indicate a significant effect after α=.05*

*SI =social interactions*
